# Associations of plasma proteomic and polygenic profiling with incident psoriasis risk: a prospective cohort study

**DOI:** 10.7189/jogh.16.04179

**Published:** 2026-05-29

**Authors:** Ting Tian, Tian Tian, Tongtong Hong, Yong He, Xiaoke Wang, Leqi Qian, Sihan Deng, Ruixin Zhou, Mingjun Jiang, Jingyi Fan, Yuancheng Li

**Affiliations:** 1 Central Research Laboratory, Jiangsu Provincial Key Laboratory of Dermatology, Hospital for Skin Diseases, Institute of Dermatology, Chinese Academy of Medical Sciences & Peking Union Medical College, Nanjing, China; 2 Institute of Occupational Disease Prevention, Jiangsu Provincial Center for Disease Control and Prevention, Nanjing, China; 3 Health Management Center, The Affiliated Suzhou Hospital of Nanjing Medical University, Suzhou Municipal Hospital, Gusu School, Nanjing Medical University, Suzhou, China

## Abstract

**Background:**

Psoriasis is a common chronic immune-mediated disease with substantial systemic and public health burden, yet early molecular signatures associated with its onset remain insufficiently characterised. Although both circulating proteins and genetic susceptibility may contribute to psoriasis development, their prospective and joint associations with incident psoriasis remain unclear. We aimed to evaluate the individual and combined effects of proteomic and polygenic profiling on psoriasis risk.

**Methods:**

We identified psoriasis-related protein signature using plasma proteomic data (2919 proteins) from 39 377 individuals in the UK Biobank. We prioritised eight biomarkers to formulate protein risk scores (ProSs) and computed polygenic risk scores (PRSs) using 62 single-nucleotide polymorphisms (SNPs). Then, we used Cox proportional hazards models to evaluate association of ProS and PRS with psoriasis risk, and performed stratified and sensitivity to explore the robustness of our findings.

**Results:**

A total of 479 incident psoriasis cases occurred over a median follow-up of 13.60 years (interquartile range = 12.89–14.31). The hazard ratios (HRs) for psoriasis were 1.40 (95% confidence interval (CI) = 1.08–1.80) and 2.72 (95% CI = 2.17–3.42) in the medium and high ProS groups, respectively, *vs*. the low ProS group (*P*-value for trend <0.001). When considering genetic susceptibility, participants with a high PRS and a high ProS had a greater risk of incident psoriasis (HR = 4.25; 95% CI = 2.97–6.07) compared to those with a low PRS and low ProS, as well as a greater 10-year absolute risk (25.99 per 1000 individuals). They also had a relative excess risk of 2.16 (95% CI = 0.98–3.35) due to additive interaction, accounting for 49% (95% CI = 29%-69%) of the psoriasis incidence. In the high genetic risk group, compared with individuals with low ProS, those with high ProS had an excess risk of 18.45 (95% CI = 17.90–18.98) cases per 1000 individuals over 10 years.

**Conclusions:**

Integrating the proteomic and polygenic tools could help improve the precision of psoriasis risk classification and facilitate the identification of high-risk populations.

Psoriasis is a common, chronic immune-mediated inflammatory skin disease affecting over 60 million people worldwide [1]. Its prevalence varies substantially from approximately 0.1% to 10% across global regions, ethnic groups, and socioeconomic settings [1–4]. The condition has systemic effects on joints, cardiovascular health, and metabolism, and it negatively impacts affected individuals’ quality of life [4,5]. Therefore, psoriasis poses a significant public health burden whose potential causative factors must be urgently identified.

Human serum and plasma are essential to clinical and biological studies, as they allow for the determination of patients’ health and disease conditions and may contain biomarkers for disease and treatment response [6–8]. Previous studies have identified a total of 129 proteins associated with psoriasis [9], while proteomic analyses have revealed some clues regarding its pathogenesis [10,11]. For example, Yu *et al*. [9] found a positive association between the psoriasis area and severity index score and the serum proteins PI3 (*r* = 0.634; *P* = 0.0009), CCL22 (*r* = 0.561; *P* = 0.0054), and IL-12B (*r* = 0.465; *P* = 0.0255) among 16 healthy controls and 23 psoriasis patients. Ryu *et al*. [10] indicated higher expression of multiple proteins such as GSTP1, SFN, and PRDX2 in psoriatic lesions in a proteomic analysis of 40 psoriasis cases and 5 healthy volunteers, suggesting dysregulation of cell proliferation, immune regulation, and inflammatory pathways. However, these studies involving proteins adopted case-control designs with a limited number of participants. In this context, a population-based longitudinal study is more appropriate way for estimating early molecular signatures associated with disease onset for risk stratification. Moreover, few studies have identified a link between protein signature and psoriasis risks.

The development of psoriasis is influenced by a variety of factors, including genetic predisposition. To date, more than 100 genetic loci have been reported to be associated with psoriasis susceptibility, explaining around 30% of the heritability of the disease [12–18]. While non-genetic factors also play an important role in psoriasis incidence and contribute to its risk stratification [19,20], previous research has usually focused on the separate effects of protein or genetic risk on psoriasis [9,20]. There have been no reports evaluating the potential combined and interactive effects of protein signature and genetic susceptibility on the risk of psoriasis.

Leveraging data from the UK Biobank, we aimed to identify psoriasis-related circulating proteomic signatures, evaluate their associations with incident psoriasis, and explore the joint effects of proteomic and genetic susceptibility on psoriasis risk.

## METHODS

We sourced our data from the UK Biobank [21], a population-based prospective cohort study (2006–2010) that collected omics and phenotypic data from over 500 000 individuals aged 40–69 years [22]. The UK Biobank study received approval from the North West Multi-Centre Research Ethics Committee, and all participants provided written informed consent.

We first identified psoriasis-related protein biomarkers in the UKB cohort. Then, we constructed a protein risk score (ProS) and a polygenic risk score (PRS) and evaluated their associations with incident psoriasis. Finally, we examined the combined effects and potential interactions between ProS and PRS on psoriasis risk (**Online Supplementary Document**).

### Proteomic measurements and participant selection

In the UK Biobank Pharma Proteomics Project, 2923 proteins were measured in 53 013 participants *via* the Olink Explore platform [23–25]. Rigorous quality control procedures were implemented, including: exclusion of participants who withdrew consent, had unprocessed data, or were identified as outliers; removal of samples and sample swaps that failed quality control; and normalisation of protein levels using log2 transformation [23,26,27].

We excluded participants with psoriasis at baseline, with cancer, and with sex discordance (*i.e.* where genetically-inferred sex did not match their self-reported sex). We further excluded samples with more than 1000 missing proteins and proteins with greater than 25% missingness. Missing proteomic values after quality control were imputed using the k-nearest neighbour method, which has been widely used in high-dimensional omics datasets [28].

### Identification of psoriasis-related proteomic signatures

We adopted a multi-step approach to identifying psoriasis-related proteomic signatures. First, we evaluated associations between each of the 2919 proteins and incident psoriasis among all 39 377 participants using Cox proportional hazards models. Second, we conducted internal replication analyses to assess robustness by excluding cases diagnosed within the first year of follow-up and by excluding participants with baseline hypertension, diabetes, dyslipidaemia, or cardiovascular disease. We controlled for multiple testing using the false discovery rate (FDR), with FDR-adjusted *P*-values <0.05 being considered statistically significant. Proteins with consistent effect directions and statistical significance across analyses were considered validated (Data S1–4 in the **Online Supplementary Document**). Third, we applied least absolute shrinkage and selection operator (LASSO) regression (‘glmnet’ package in *R*) [29] to the validated proteins to select representative biomarkers using an L1-norm penalty. The penalty parameter (*λ*) was selected using 10-fold cross-validation based on the one-standard-error rule to improve model stability. We then calculated the ProS as follows:

*ProS* = 0.0404 × *ALDH3A1* + 0.0673 × *CCL22* + 0.0113 × *CDCP1* + 0.0591 × *CTSD* + 0.0554 × *DEFB4A_DEFB4B* + 0.2128 × *IL19* + 0.1837 × *PI3* + 0.0299 × *PLA2G15*

The ProS was categorised into three groups by quartiles: low (quartiles 1–2), medium (quartile 3), and high (quartile 4).

### PRS construction

The UK Biobank team performed genotyping, imputation, and quality control of single-nucleotide polymorphisms (SNPs) [22]. Here, we constructed a PRS using 62 psoriasis-associated SNPs identified from previous genome-wide association studies [16,20,30] (Data S5 in the **Online Supplementary Document**) using the following formula:

*PRS* = *β*_1_ × *SNP*_1_ + *β*_2_ × *SNP*_2_ + …. + *β_n_* × *SNP_n_*

Here, *n* represents the number of SNPs; SNP values were coded as 0, 1, or 2; and *β* represents the per-allele log odds ratio for psoriasis associated with each SNP. Participants were categorised into three genetic risk groups based on PRS tertiles: low (tertile 1), intermediate (tertile 2), and high (tertile 3).

### Covariates and outcome ascertainment

Baseline information on age, gender, ethnicity, Townsend deprivation index (a measure of socioeconomic status), occupation, body mass index (BMI), smoking status, and alcohol consumption was retrieved from the UK Biobank.

Prevalent comorbidities, including hypertension, diabetes, dyslipidaemia, and cardiovascular disease, were identified using self-reported information, primary care data, and hospital inpatient records from the UK Biobank (Data S6 and S7 in the **Online Supplementary Document**). We selected the covariates based on prior literature and biological plausibility as potential confounders of the associations between circulating proteins and psoriasis risk [31]. Incident psoriasis was identified through linked electronic health records within the UK Biobank, including hospital inpatient data, primary care records, and death registry data, using ICD-9/ICD-10 codes, Read Codes Version 2, and Read Codes Clinical Terms Version 3 (Data S6 in the **Online Supplementary Document**).

Participants were followed from baseline until the first diagnosis of psoriasis, death, loss to follow-up, or the end of available registry data (31 October 2022 for England, 31 August 2022 for Scotland, and 31 May 2022 for Wales), whichever occurred first. The follow-up duration was determined by the availability of longitudinal health records and was sufficient to capture incident psoriasis cases and evaluate long-term associations between protein levels, genetic susceptibility, and psoriasis risk.

### Statistical analysis

We summarised baseline characteristics as means (x̄) and standard deviations (SDs) for continuous variables, and counts and percentages for categorical variables. Follow-up time was summarised using median and interquartile ranges (IQRs), as its distribution was not assumed to be normal. We used Cox proportional hazards models to estimate hazard ratios (HRs) and 95% confidence intervals (CIs) for the associations of ProS and PRS with incident psoriasis. We tested the proportional hazards assumption using Schoenfeld residuals and observed no substantial deviations. Participants with missing covariate data were excluded from regression analyses.

We analysed ProS and PRS both as continuous variables (per SD increase) and as categorical variables based on their distributions to facilitate interpretation of risk differences across clinically relevant groups and to explore potential nonlinear associations. We used three models: model 0, which was the unadjusted model; model 1, which was adjusted for age, gender, assessment centre, ethnicity, occupation, and Townsend deprivation index, and model 2, which was further adjusted for BMI, smoking, and drinking status. We assessed nonlinear relationships *via* restricted cubic splines and used Kaplan–Meier curves with log-rank tests evaluated risk stratification.

We conducted interaction analyses to explore potential joint associations between ProS and PRS. We tested multiplicative interaction *via* product terms and quantified additive interaction using the relative excess risk due to interaction and attributable proportion due to interaction. We derived the predicted 10-year absolute risk from the fitted Cox proportional hazards models using the baseline cumulative hazard at 10 years and individual linear predictors. We summarised absolute risks within each group and expressed them as the number of expected cases per 1000 individuals over 10 years. Corresponding 95% CIs were obtained from 1000 bootstrap resamples.

We performed subgroup analyses by gender, age, BMI, ethnicity, occupation, smoking, and drinking status. We assessed effect modification by including multiplicative interaction terms between the risk scores and subgroup variables in the Cox proportional hazards models. Heterogeneity across subgroups was evaluated using Cochran’s Q test and *I^2^* statistics. We further conducted sensitivity analyses by excluding psoriasis cases diagnosed within the first year of follow-up; excluding cases diagnosed within the first three years; excluding participants with baseline comorbidities; retaining original proteomic data without imputation. We conducted all analyses in *R*, version 4.3.2 (R Core Team, Vienna, Austria). Two-sided *P*-values <0.05 were considered statistically significant.

## RESULTS

### Study participants

We excluded 1371 participants with psoriasis at baseline, 4913 with cancer, and 180 with sex discordance from the initial cohort (Figure S2 in the **Online Supplementary Document**). We further excluded 7172 samples with more than 1000 missing proteins and removed 4 proteins with greater than 25% missingness. This resulted in a final analytic cohort of 39 377 participants with measurements for 2919 proteins. 

We included 39 377 participants with proteomic data, among whom 479 incident cases of psoriasis were recorded over a median follow-up period of 13.60 years (interquartile range = 12.89–14.31) (**Table 1**). Compared with participants without psoriasis, individuals with incident psoriasis had a higher Townsend deprivation index (x̄ = −1.15, SD = 3.20 *vs*. x̄ = −0.84, SD = 3.36), but a lower employment rate (85.59% *vs*. 90.85%). They also had a higher BMI (x̄ = 28.61, SD = 5.49 *vs*. x̄ = 27.45, SD = 4.79), a higher prevalence of current smoking (15.24% *vs*. 10.70%), and a higher incidence of comorbidities such as diabetes (7.93% *vs*. 5.58%), dyslipidaemia (20.67% *vs*. 16.72%), and cardiovascular disease (12.32% *vs*. 9.04%).

**Table 1:** Participants’ baseline characteristics*

	Total (n = 39 377)	Non-psoriasis (n = 38 898)	Psoriasis (n = 479)	*P*-value
**Age in years, x̄ (SD)**	56.49 (8.22)	56.48 (8.22)	57.03 (7.87)	0.131
**Gender**				0.903
Female	21 030 (53.41)	20 776 (53.41)	254 (53.03)	
Male	18 347 (46.59)	18 122 (46.6)	225 (46.97)	
**Assessment center**				0.008
England	34 760 (88.27)	34 352 (88.31)	408 (85.18)	
Scotland	2930 (7.44)	2893 (7.44)	37 (7.72)	
Wales	1687 (4.28)	1653 (4.25)	34 (7.10)	
**Ethnicity**				0.066
White	36 404 (92.45)	35 948 (92.42)	456 (95.20)	
Other	2827 (7.18)	2805 (7.21)	22 (4.59)	
Unknown/missing	146 (0.37)	145 (0.37)	1 (0.21)	
**Occupation**				<0.001
Unemployed	3187 (8.09)	3125 (8.03)	62 (12.94)	
Employed	35 749 (90.79)	35 339 (90.85)	410 (85.59)	
Unknown/missing	441 (1.12)	434 (1.12)	7 (1.46)	
**Townsend deprivation index, x̄ (SD)**	−1.15 (3.20)	−1.15 (3.20)	-0.84 (3.36)	0.045
**BMI in kg/m^2^, x̄ (SD)**	27.46 (4.80)	27.45 (4.79)	28.61 (5.49)	<0.001
**Smoking status**				<0.001
Never	21 510 (54.63)	21 311 (54.79)	199 (41.54)	
Former	13 443 (34.14)	13 237 (34.03)	206 (43.01)	
Current	4235 (10.76)	4162 (10.70)	73 (15.24)	
Unknown/missing	189 (0.48)	188 (0.48)	1 (0.21)	
**Drinking status**				0.039
Never	1893 (4.82)	1875 (4.83)	18 (3.77)	
Former	1492 (3.80)	1462 (3.77)	30 (6.28)	
Current	35 890 (91.38)	35 460 (91.40)	430 (89.96)	
Unknown/missing	102 (0.26)	101 (0.26)	1 (0.21)	
**Comorbidities**				
Hypertension	11 466 (29.12)	11 310 (29.08)	156 (32.57)	0.105
Diabetes	2210 (5.61)	2172 (5.58)	38 (7.93)	0.034
Dyslipidaemia	6603 (16.77)	6504 (16.72)	99 (20.67)	0.025
Cardiovascular disease	3575 (9.08)	3516 (9.04)	59 (12.32)	0.016

BMI – body mass index, SD – standard deviation, x̄ – mean

*Presented as n (%) unless specified otherwise.

### Protein signatures of psoriasis

A total of 456 of 2919 proteins were significantly associated with incident psoriasis in the main analysis. After excluding 127 cases occurring within the first year of follow-up, 368 proteins remained significantly associated with incident psoriasis. Excluding 14 958 participants with baseline comorbidities (hypertension, diabetes, dyslipidaemia, or cardiovascular disease) left 160 significant proteins. Ultimately, 136 proteins were consistently associated (all post-FDR *P*-values <0.05) with psoriasis risk across all analyses (Data S1–4 in the **Online Supplementary Document**).

Eight of these 136 proteins (ALDH3A1, CCL22, CDCP1, CTSD, DEFB4A_DEFB4B, IL19, PI3, and PLA2G15) were selected using LASSO modelling to derive psoriasis proteomic signatures. Among these, PLA2G15 had a higher risk association (HR = 1.87; 95% CI = 1.40–2.51) after adjusting for age, gender, assessment centre, ethnicity, occupation, Townsend deprivation index, BMI, smoking, and drinking status. The associations between individual proteins and psoriasis risk displayed either dose-response or J-shaped patterns (Figures S3 and S4 in the **Online Supplementary Document**).

### Association of ProS with incident psoriasis risk

**Figure 1:**
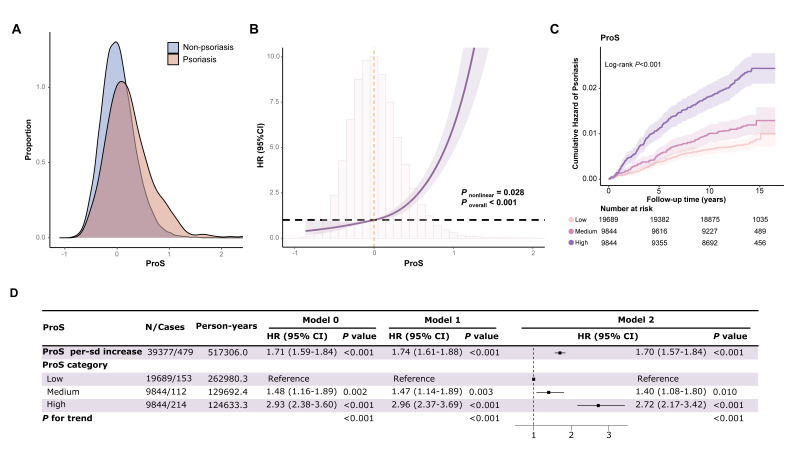
Association of ProS with incident psoriasis risks. **Panel A.** Distribution of ProS in participants affected with or without psoriasis. **Panel B.** Nonlinear relationships between ProS and psoriasis risks. **Panel C.** Kaplan-Meier cumulative incidence curves across risk groups based on ProS. **Panel D.** Association effects between ProS in low, medium, and high ProS groups and incident psoriasis risks. Model 0 was unadjusted. Model 1 was adjusted for age, gender, assessment center, ethnicity, occupation, and Townsend deprivation index. Model 2 was further adjusted for BMI, smoking status, and drinking status. CI – confidence interval, HR – hazard ratio, ProS – protein risk score.

We observed a significantly higher distribution of the ProS in incident psoriasis cases compared to participants without psoriasis. Through the restricted cubic spline model, we identified a reverse J-shaped association between ProS and psoriasis risk (*P*-value for nonlinearity = 0.028, *P*-value for overall association <0.001). We observed that each per-SD increase in ProS is significantly connected to an increased likelihood of psoriasis in the crude model (HR = 1.71; 95% CI = 1.59–1.84, *P* < 0.001) and full adjusted model (HR = 1.70; 95% CI = 1.57–1.84, *P* < 0.001). For the J-shaped association, we subsequently divided the ProS into quartiles and re-categorised them into three levels: low, medium, and high. The Kaplan-Meier cumulative incidence curves (**Figure 1**, Panel C) showed a clear separation between the three risk groups (log-rank *P* < 0.001), with the highest risk of psoriasis observed in the high ProS group. Compared with participants in the low ProS group, those in the medium and high ProS groups had a significantly higher risk of psoriasis, with HRs of 1.40 (95% CI = 1.08–1.80, *P* = 0.010) for the medium group and 2.72 (95% CI = 2.17–3.42, *P* < 0.001) for the high group in model 2, respectively (*P*-value for trend <0.001). Stratified analyses showed that the association of ProS was greater among unemployed participants (*P*-value for heterogeneity = 0.006, *P*-value for interaction = 0.050) (**Figure 1**, Panels A–D; Table S1 in the **Online Supplementary Document**).

### Association of PRS with incident psoriasis risk

**Figure 2:**
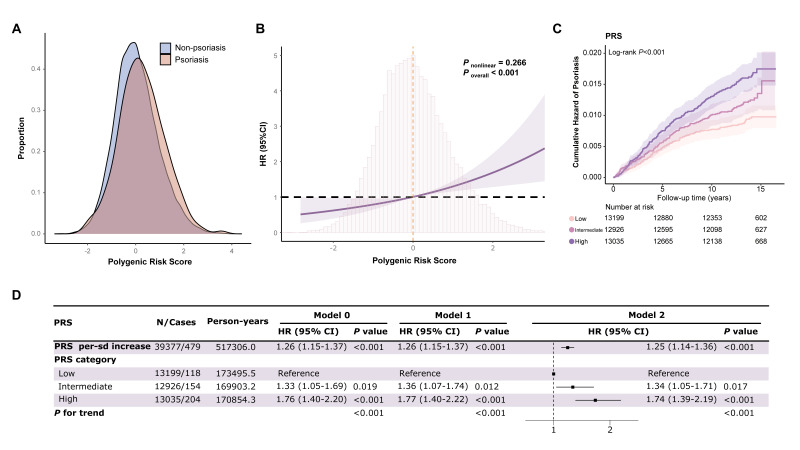
Association of PRS with incident psoriasis risks. **Panel A.** Distribution of PRS in participants affected with or without psoriasis. **Panel B.** Nonlinear relationships between PRS and psoriasis risks. **Panel C.** Kaplan-Meier cumulative incidence curves across risk groups based on PRS. **Panel D.** Association effects between PRS in low, intermediate, and high PRS groups and incident psoriasis risks. Model 0 was unadjusted. Model 1 was adjusted for age, gender, assessment center, ethnicity, occupation, and Townsend deprivation index. Model 2 was further adjusted for BMI, smoking status, and drinking status. CI – confidence interval, HR – hazard ratio, PRS – polygenic risk score.

Density plots demonstrated a clear shift in the PRS distribution toward higher values among psoriasis cases compared to those without psoriasis. There wasa almost no correlation between each protein and the PRS (Spearman’s *r*<0.05). Kaplan–Meier cumulative incidence curves for three PRS risk groups (**Figure 2**, Panel C) showed a clear separation between groups (log-rank *P* < 0.001), with the high PRS group having the highest risk of psoriasis. We observed a positive association between genetic susceptibility and the risk of incident psoriasis. The restricted cubic spline curve revealed a dose-response relationship between PRS and the increased risk of psoriasis (*P*-value for nonlinearity = 0.266, *P*-value for overall association <0.001). In the model adjusted for age, gender, assessment centre, ethnicity, occupation, Townsend deprivation index, BMI, smoking status, and drinking status, the HRs for psoriasis were 1.34 (95% CI = 1.05–1.71) for those with intermediate genetic risk and 1.74 (95% CI = 1.39–2.19) for those with high genetic risk, respectively (*P*-value for trend <0.001), compared with individuals at low genetic risk for psoriasis. For each per-SD increase in PRS, the risk of psoriasis increased by 25% (HR = 1.25, 95% CI = 1.14–1.36). Stratified analyses showed that the effect of PRS on psoriasis was significantly larger in unemployed individuals (*P*-value for heteorgeneity = 0.020) or smokers (*P*-value for heteorgeneity = 0.007). Both occupational status (*P*-value for interaction = 0.017) and smoking status (*P*-value for interaction = 0.007) significantly interacted multiplicatively with PRS regarding the risk of developing psoriasis (**Figure 2**, Panels A–D; Figure S5 and Table S2 in the **Online Supplementary Document**).

### Combined effect and interaction of ProS and PRS on incident psoriasis risk

**Figure 3:**
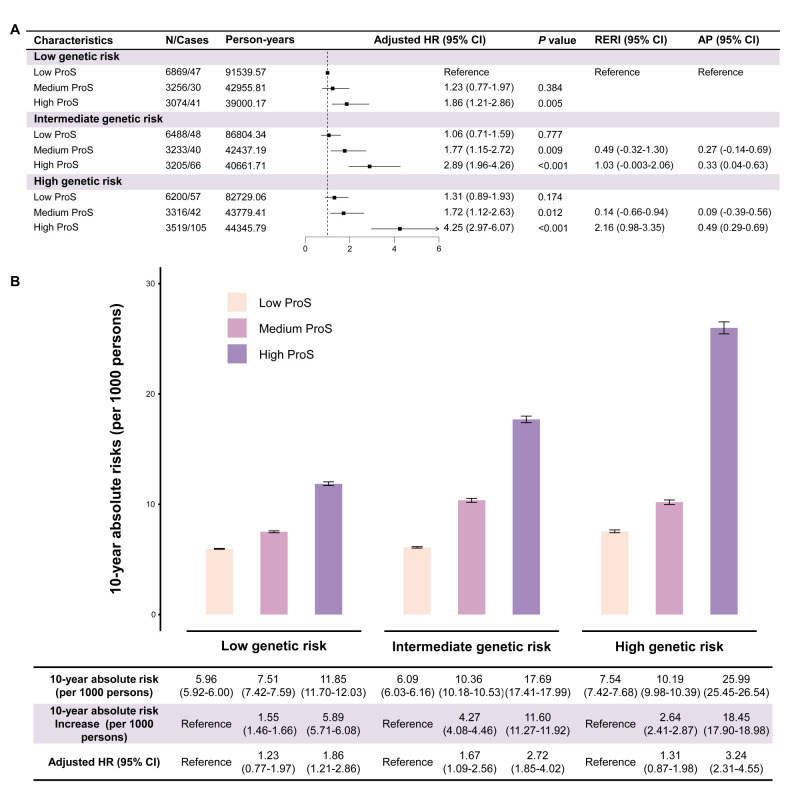
Combined associations of ProS and genetic risks with incident psoriasis. **Panel A.** Associations between ProS and risk of incident psoriasis stratified by genetic risk. **Panel B.** Ten-year absolute risks and risk increase of incident psoriasis according to ProS within each genetic risk category. The HRs were estimated using Cox proportional hazards regression by model 2, with adjustment for age, gender, assessment center, ethnicity, occupation, Townsend deprivation index, BMI, smoking status, drinking status. The low ProS and the low PRS groups were the reference categories for estimating the RERI and AP. AP – attributable proportion due to the interaction, CI – confidence interval, HR – hazard ratio, PY – person-years, ProS – protein risk score, RERI – relative excess risk due to the interaction.

We estimated the combined effects of protein signature and genetic susceptibility on psoriasis with model 0 and model 1, where we also observed that the likelihood of psoriasis increased with higher levels of both the ProS and the PRS. Compared with participants who had both low ProS and low PRS, those with high ProS and high PRS had a higher risk of psoriasis (HR = 4.25; 95% CI = 2.97–6.07, *P* < 0.001). Similarly, we observed a higher risk across different subgroups: the HRs were 3.69 (95% CI = 2.08–6.54) for males, 4.68 (95% CI = 2.94–7.46) for females, 3.95 (95% CI = 2.49–6.27) for individuals aged <60 years, and 4.87 (95% CI = 2.76–8.59) for those aged ≥60 years. We observed positive joint associations in the additional interaction scale between ProS and PRS on psoriasis risk. Specifically, participants with high levels of ProS and high PRS had a relative excess risk of 2.16 (95% CI = 0.98–3.35) due to additive interaction, accounting for 49% (95% CI = 29–69%) of the psoriasis incidence (**Figure 3**, Panel A; Tables S3–5 in the **Online Supplementary Document**).

We observed a significant effect in the relative risk increase of incident psoriasis from ProS across genetic risk groups (**Figure 3**, Panel B). Individuals with high ProS in the high genetic risk group showed the highest standardised 10-year absolute risk, with the risk of 25.99 (95% CI = 25.45–26.54) per 1000 persons. Compared with group of low ProS, the standardised 10-year absolute risk increase across low, intermediate, and high genetic risk categories were 5.89 (95% CI = 5.71–6.08), 11.60 (95% CI = 11.27–11.92), and 18.45 (95% CI = 17.90–18.98) per 1000 persons when individuals also had a high ProS.

After limiting the sensitivity analyses to participants with a follow-up period >1 year or >3 years, the combined effect of ProS and PRS on the risk of incident psoriasis was consistent with the main analysis. The results also remained stable when participants who had a baseline history of hypertension, diabetes, dyslipidaemia or cardiovascular disease were excluded. Sensitivity analyses restricted to the original proteomic data without imputation yielded similar results (Table S6–9 in the **Online Supplementary Document**).

## DISCUSSION

Using a multi-step strategy, we identified circulating proteomic signatures associated with psoriasis and prioritised eight protein biomarkers to create a ProS. Both individual proteins and ProS were significantly correlated with psoriasis risk. Additionally, the PRS, reflecting genetic susceptibility, showed a positive dose-response relationship with psoriasis risk. In this large-scale prospective cohort study, we assessed how ProS affects the likelihood of incident psoriasis, while considering the modifying effect of genetic risk. Individuals with high genetic risk and high ProS had a significantly higher risk of incident psoriasis compared to those with low genetic risk and low ProS. These findings may suggest potential joint associations between proteomic and genetic susceptibility in relation to psoriasis risk.

Psoriasis is primarily driven by immune responses, and proteins are key players in inflammation and immunity, whereby they serve as as important biomarkers. To mitigate potential false-positive findings in this high-dimensional proteomic analysis, we applied a multi-step analytical strategy including replication analyses, FDR correction, and LASSO regression. We established a risk score for psoriasis based on eight plasma proteins from the UK Biobank: ALDH3A1, CCL22, CDCP1, CTSD, DEFB4A_DEFB4B, IL19, PI3, and PLA2G15. Previous studies have shown that PI3 and CCL22 correlate with the Psoriasis Area and Severity Index score (PI3: *r* = 0.634, *P* = 0.0009; CCL22: *r* = 0.561, *P* = 0.0054) [9]. PI3, absent in normal skin, but highly expressed in psoriatic keratinocytes [32], is up-regulated in psoriatic skin samples [33]. CCL22 attracts immune cells to inflammatory sites and its expression in psoriatic skin correlates with a good response to infliximab therapy [34]. ALDH3A1 is highly expressed in psoriasis vulgaris [35], while CDCP1 is elevated in psoriatic arthritis (*P* < 0.001) [36]. Previous research has shown that CTSD influences psoriasis pathogenesis by modulating macrophage activity and mediating inflammatory responses [37]. In addition, IL-19 and IL-36 are implicated in psoriasis pathogenesis [38–40], with IL-36 stimulating antimicrobial peptide production (DEFB4A-DEFB4B) in target cells [41]. However, PLA2G15 among these proteins has not been previously linked to psoriasis, suggesting a potential area for future research to explore its underlying mechanisms.

The association of ProS with psoriasis significantly varied by genetic variants. So far, more than 100 psoriasis loci have been successfully identified by GWAS [12–18]. However, these loci can only explain around 30% of psoriasis heritability. Here, we further validated an additive interaction between high genetic risk and high ProS on psoriasis incidence in a low ProS setting (relative excess risk due to interaction = 2.16; 95% CI = 0.98–3.35; attributable proportion due to interaction = 49%, 95% CI = 29–69). While genetic factors are unavoidable, our findings suggest that individuals with high levels of ProS experience a greater increase of 10-year absolute risk (25.99 per 1000 individuals).

The strengths of our study, which is the first investigation into the association of plasma proteomic and polygenic profiling with the psoriasis risks, lie in its large sample size and prospective design. We further examined the combined and interactive effects of plasma protein and genetic susceptibility on psoriasis. However, several limitations should be acknowledged. First, residual confounding cannot be completely excluded, despite our adjustment for multiple variables, and reverse causation may be possible. Second, psoriasis cases were identified using linked electronic health records within the UK Biobank, so some degree of outcome misclassification cannot be completely excluded. Third, although psoriasis-related proteomic biomarkers were identified through a multi-step analytical approach, external validation in independent populations will be necessary before potential clinical implementation. Fourth, we handled missing proteomic values using k-nearest neighbour imputation and addressed missing covariate data using complete-case analysis, which may introduce bias. Finally, although we applied LASSO regression to reduce model complexity and mitigate potential overfitting, the derived protein risk score requires further validation in independent data sets to confirm its robustness and reproducibility.

Our findings may have potential implications for risk stratification and early identification of individuals at elevated risk of psoriasis. The integration of circulating protein biomarkers with genetic susceptibility measures may improve the identification of high-risk populations and facilitate targeted prevention strategies. In clinical settings, such approaches could help identify individuals who may benefit from closer dermatological monitoring or early lifestyle interventions. From a public health perspective, the combination of proteomic and genetic risk information may support more personalised prevention strategies.

## CONCLUSIONS

In this large prospective cohort study, we found that higher proteomic risk scores were associated with an increased risk of incident psoriasis. Individuals with both high proteomic risk and high genetic susceptibility had the greater risk of developing psoriasis. Our analysis of the combined effects and additive interactions between the plasma protein and genetic susceptibility suggests that integrating these assessment approach can enhance the precision of psoriasis risk classification and facilitate the identification of high-risk populations.

## Additional material


Online Supplementary Document
