# Supplementary material for: Associations of plasma proteomic and polygenic profiling with incident psoriasis risk: a prospective cohort study
Source: J Glob Health. 2026 May 29;16:04179. doi: 10.7189/jogh.16.04179 (PMC13219973; doi:10.7189/jogh.16.04179)
Supplement: Online Supplementary Document [file jogh-16-04179-s001.zip › jogh-16-04179-s001.pdf]

Supplement to: Tian T, Tian T, Hong T, He Y, Wang X, Qian L, Deng S, Zhou R, Jiang M, Fan J, Li Y. Associations of plasma proteomic and polygenic profiling with incident psoriasis risk: a prospective cohort study. J Glob Health. 2026;16:04179.

Figure S1. Schematic overview of the study design.

Figure S2. Flow chart for selecting the study participants.

Figure S3. Lasso-Cox regression analysis of plasma proteins with incident psoriasis risks.

Figure S4. Non-linear relationships between each protein and psoriasis risks.

Figure S5. Spearman's correlation matrix among proteins and PRS.

Table S1. Associations between protein risk score (ProS) and incident psoriasis risks stratified according to different subgroups.

Table S2. Associations between polygenic risk score (PRS) and incident psoriasis risks stratified according to different subgroups.

Table S3. Combined associations of protein risk score (ProS) and genetic risks with incident psoriasis.

Table S4. Combined associations of protein risk score (ProS) and genetic risks with psoriasis among males and females.

Table S5. Combined associations of protein risk score (ProS) and genetic risks with psoriasis among different age groups.

Table S6. Sensitivity analyses after excluding incident cases during the first year of follow-up.

Table S7. Sensitivity analyses after excluding incident cases during the first 3 years of follow-up.

Table S8. Sensitivity analyses after excluding participants with hypertension, diabetes, dyslipidemia or cardiovascular disease at baseline.

Table S9. Sensitivity analyses in the original proteomic data without imputation.

Table S10. Outline of JoGH's Guidelines for Reporting Analyses of Big Data Repositories Open to the Public (GRABDROP) items

Text S1. Explanation of authorship change statement

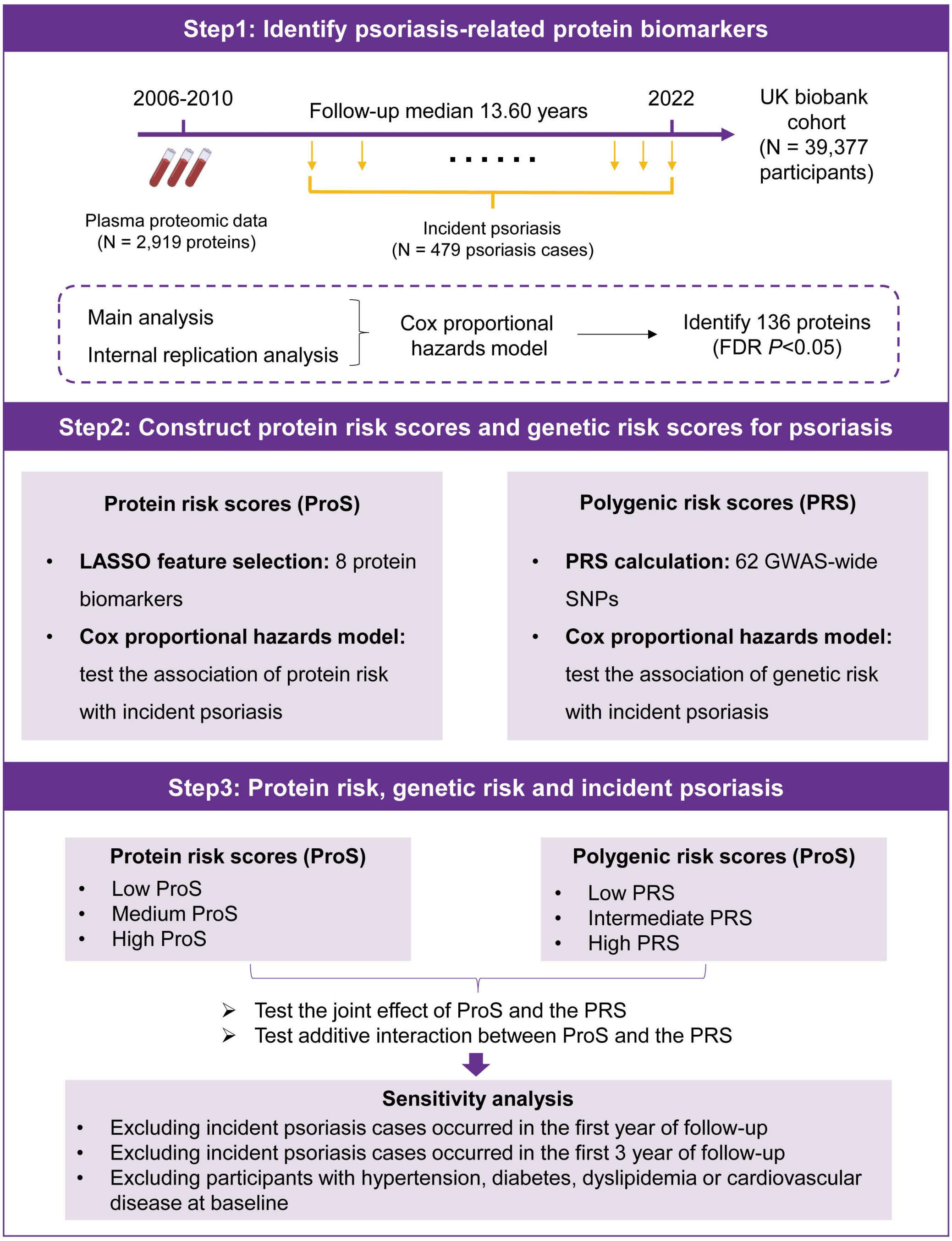

Figure S1. Schematic overview of the study design.

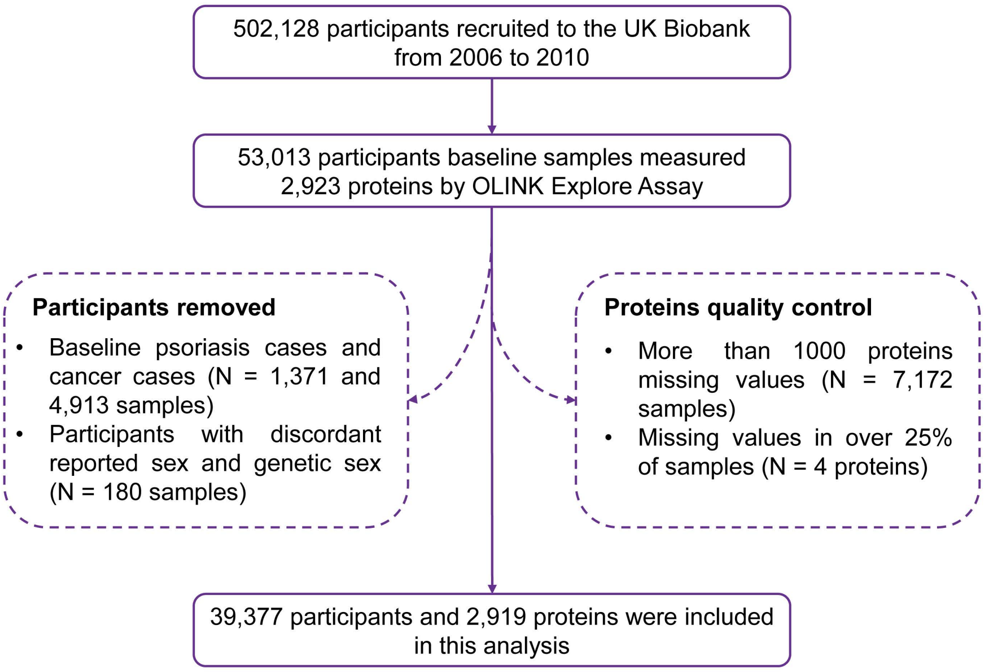

Figure S2. Flow chart for selecting the study participants.

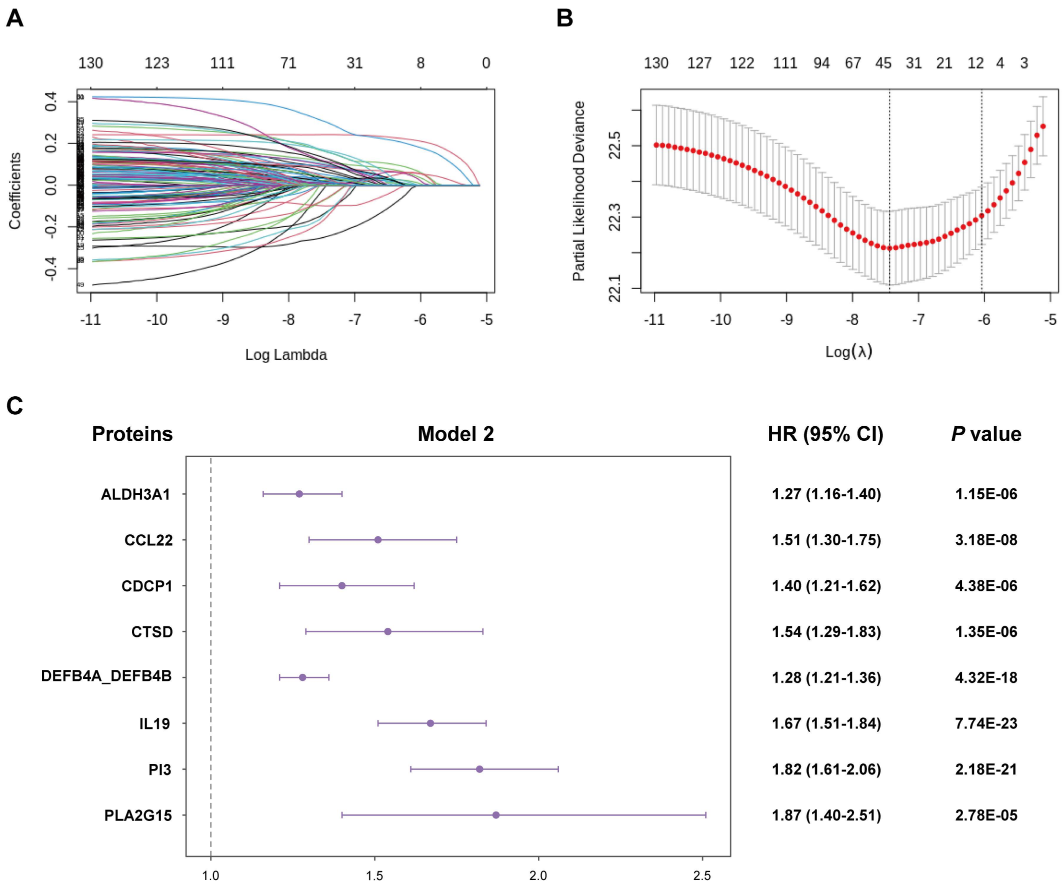

Figure S3. Lasso-Cox regression analysis of plasma proteins with incident psoriasis risks. (A) The Lasso coefficient profile of 136 plasma proteins. (B) The tuning parameters (log λ) of plasma proteins were selected to cross-verify the error curve. The perpendicular imaginary lines were drawn at the optimal value based on the minimal criterion and 1-se criterion. (C) The results of 8 plasma proteins that used to construct protein risk score (ProS) with psoriasis from Cox proportional hazards regression. The HR (95% CI) were estimated using Cox proportional hazard models. The adjusted covariates were age, gender, assessment center, ethnicity, occupation, Townsend deprivation index, BMI, smoking status, drinking status. HR, hazard ratio; 95% CI, 95% confidence interval.

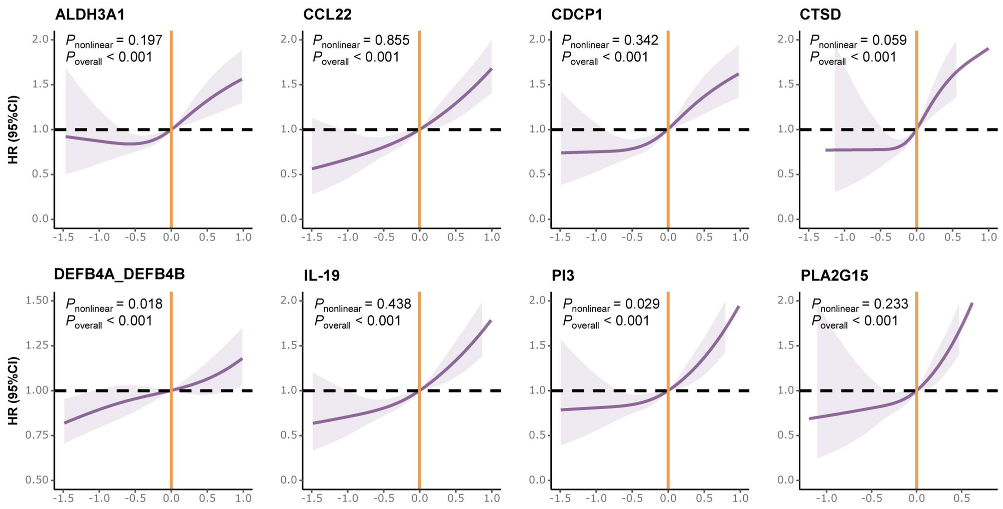

Figure S4. Non-linear relationships between each protein and psoriasis risks.

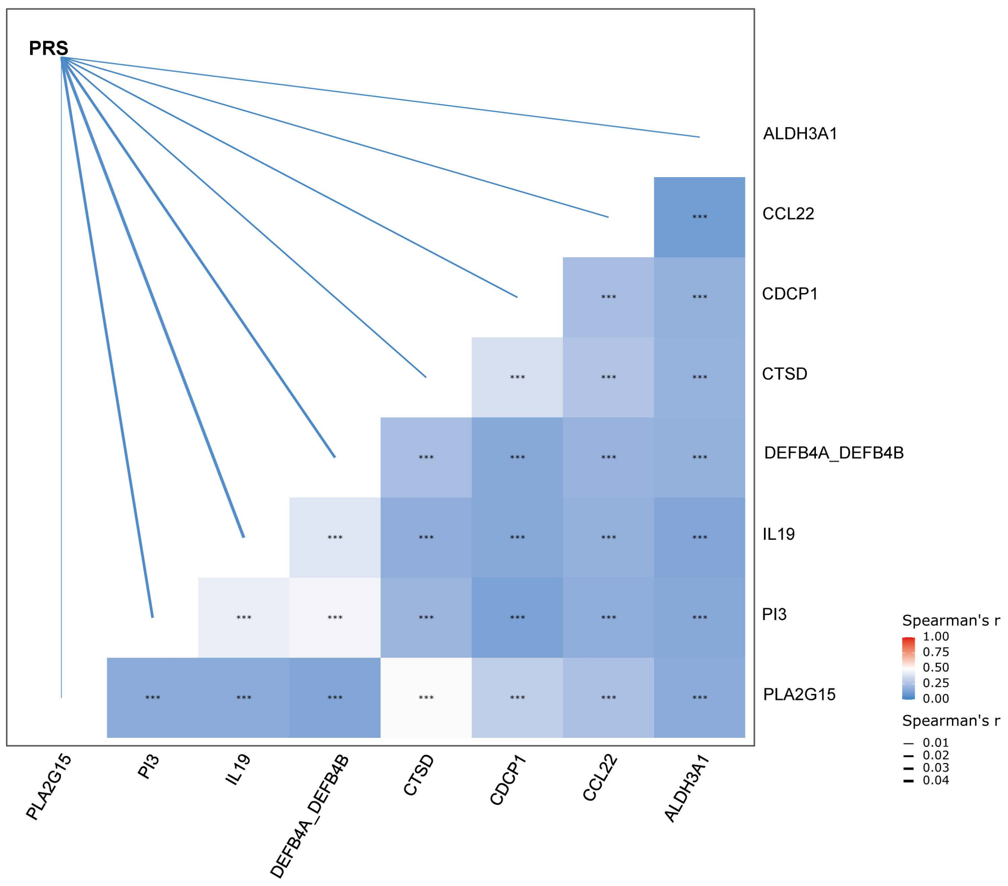

Figure S5. Spearman's correlation matrix among proteins and PRS. The correlation map displays the Spearman's correlation coefficients between each protein and the PRS.

Table S1. Associations between protein risk score (ProS) and incident psoriasis risks stratified according to different subgroups.

Subgroups	N/Cases	HR (95% CI)	*P* value	*P* for heterogeneity	*P* for interaction*
**Gender**					
Female	21030/254	1.57 (1.39-1.77)	<0.001	0.052	0.076
Male	18347/225	1.84 (1.65-2.06)	<0.001		
**Age**					
<60	22328/262	1.73 (1.55-1.92)	<0.001	0.701	0.342
⩾60	17049/217	1.67 (1.48-1.89)	<0.001		
**BMI**					
<25	12757/129	1.57 (1.33-1.86)	<0.001	0.302	0.422
⩾25	26425/349	1.74 (1.59-1.91)	<0.001		
**Ethnicity**					
White Race	36404/456	1.71 (1.57-1.86)	<0.001	0.503	0.619
Others	2827/22	1.48 (0.98-2.23)	0.062		
**Occupation**					
Unemployed	3187/62	2.19 (1.81-2.65)	<0.001	**0.006**	**0.050**
Employed	35749/410	1.62 (1.49-1.78)	<0.001		
**Smoking status**					
No	21510/199	1.53 (1.33-1.75)	<0.001	0.042	0.123
Yes	17678/279	1.82 (1.64-2.01)	<0.001		
**Drinking status**					
No	1893/18	1.74 (1.20-2.51)	0.003	0.899	0.548
Yes	37382/460	1.70 (1.56-1.84)	<0.001		

Abbreviations: BMI, body mass index; HR, hazard ratio; 95% CI, 95% confidence interval.

The HR (95% CI) were estimated using Cox proportional hazard models. The adjusted covariates were age, gender, assessment center, ethnicity, occupation, Townsend deprivation index, BMI, smoking status, drinking status.

*The multiplicative interaction was tested by introducing a product term of the two variables examined in the models.

Table S2. Associations between polygenic risk score (PRS) and incident psoriasis risks stratified according to different subgroups.

Subgroups	N/Cases	HR (95% CI)	*P* value	*P* for heterogeneity	*P* for interaction*
**Gender**					
Female	21030/254	1.28 (1.12-1.46)	<0.001	0.976	0.964
Male	18347/225	1.27 (1.11-1.47)	<0.001		
**Age**					
<60	22328/262	1.22 (1.07-1.39)	0.002	0.334	0.364
⩾60	17049/217	1.34 (1.17-1.55)	<0.001		
**BMI**					
<25	12757/129	1.48 (1.23-1.77)	<0.001	0.066	0.068
⩾25	26425/349	1.21 (1.08-1.36)	<0.001		
**Ethnicity**					
White Race	36404/456	1.27 (1.16-1.40)	<0.001	0.756	0.717
Others	2827/22	1.41 (0.76-2.60)	0.275		
**Occupation**					
Unemployed	3187/62	1.69 (1.31-2.18)	<0.001	**0.020**	**0.017**
Employed	35749/410	1.22 (1.10-1.35)	<0.001		
**Smoking status**					
No	21510/199	1.08 (0.93-1.27)	0.299	**0.007**	**0.007**
Yes	17678/279	1.42 (1.26-1.61)	<0.001		
**Drinking status**					
No	1893/18	0.84 (0.48-1.47)	0.537	0.135	0.144
Yes	37382/460	1.30 (1.18-1.43)	<0.001		

Abbreviations: BMI, body mass index; HR, hazard ratio; 95% CI, 95% confidence interval.

The HR (95% CI) were estimated using Cox proportional hazard models. The adjusted covariates were age, gender, assessment center, ethnicity, occupation, Townsend deprivation index, BMI, smoking status, drinking status.

*The multiplicative interaction was tested by introducing a product term of the two variables examined in the models.

Table S3. Combined associations of protein risk score (ProS) and genetic risks with incident psoriasis.

Characteristics	N/Cases	Person-years	Model 0		Model 1	
			HR (95% CI)	*P* value	HR (95% CI)	*P* value
**Low genetic risk**						
Low ProS	6869/47	91539.57	Ref		Ref	
Medium ProS	3256/30	42955.81	1.36 (0.86-2.15)	0.191	1.31 (0.82-2.09)	0.265
High ProS	3074/41	39000.17	**2.03 (1.34-3.09)**	**0.001**	**2.04 (1.33-3.14)**	**0.001**
**Intermediate genetic risk**						
Low ProS	6488/48	86804.34	1.08 (0.72-1.61)	0.716	1.09 (0.73-1.64)	0.659
Medium ProS	3233/40	42437.19	**1.83 (1.20-2.79)**	**0.005**	**1.86 (1.21-2.85)**	**0.004**
High ProS	3205/66	40661.71	**3.14 (2.16-4.56)**	**<0.001**	**3.21 (2.19-4.70)**	**<0.001**
**High genetic risk**						
Low ProS	6200/57	82729.06	1.34 (0.91-1.98)	0.135	1.32 (0.89-1.94)	0.163
Medium ProS	3316/42	43779.41	**1.86 (1.23-2.83)**	**0.003**	**1.86 (1.22-2.84)**	**0.004**
High ProS	3519/105	44345.79	**4.57 (3.24-6.45)**	**<0.001**	**4.65 (3.27-6.62)**	**<0.001**

Abbreviations: ProS, protein risk score; HR, hazard ratio; 95% CI, 95% confidence interval.

Model 0: adjusted for no covariates;

Model 1: adjusted for age, gender, assessment center, ethnicity, occupation, and Townsend deprivation index.

Table S4. Combined associations of protein risk score (ProS) and genetic risks with psoriasis among males and females.

Characteristics	Males			Females		
	**Incidence/ 100000 PYs**	**HR (95% CI)**	***P* value**	**Incidence/ 100000 PYs**	**HR (95% CI)**	***P* value**
**Low genetic risk**						
Low ProS	48.95	Reference		52.55	Reference	
Medium ProS	55.42	0.98 (0.46-2.09)	0.949	87.19	1.44 (0.79-2.64)	0.234
High ProS	104.89	1.86 (0.98-3.53)	0.059	105.58	**1.68 (0.89-3.17)**	**0.113**
**Intermediate genetic risk**						
Low ProS	45.05	0.87 (0.41-1.85)	0.715	60.40	1.15 (0.71-1.86)	0.560
Medium ProS	91.28	1.78 (0.92-3.46)	0.089	97.78	1.65 (0.92-2.95)	0.091
High ProS	155.25	**2.68 (1.47-4.89)**	**0.001**	175.41	**2.91 (1.70-4.96)**	**<0.001**
**High genetic risk**						
Low ProS	58.44	1.20 (0.60-2.44)	0.606	74.07	1.35 (0.85-2.15)	0.202
Medium ProS	79.21	1.46 (0.73-2.92)	0.287	113.98	**1.89 (1.10-3.25)**	**0.020**
High ProS	202.92	**3.69 (2.08-6.54)**	**<0.001**	301.25	**4.68 (2.94-7.46)**	**<0.001**

Abbreviations: ProS, protein risk score; PYs, person-years; HR, hazard ratio; 95% CI, 95% confidence interval.

The HR (95% CI) were estimated using Cox proportional hazard models. The adjusted covariates were age, gender, assessment center, ethnicity, occupation, Townsend deprivation index, BMI, smoking status, drinking status.

Table S5. Combined associations of protein risk score (ProS) and genetic risks with psoriasis among different age groups.

Characteristics	Age <60 years			Age ≥60 years		
	**Incidence/ 100000 PYs**	**HR (95% CI)**	***P* value**	**Incidence/ 100000 PYs**	**HR (95% CI)**	***P* value**
**Low genetic risk**						
Low ProS	53.39	Reference		48.09	Reference	
Medium ProS	54.64	0.89 (0.46-1.75)	0.745	88.72	1.79 (0.90-3.55)	0.098
High ProS	147.95	2.38 (1.40-4.04)	0.001	61.86	1.31 (0.62-2.77)	0.486
**Intermediate genetic risk**						
Low ProS	51.33	0.93 (0.55-1.57)	0.789	62.00	1.32 (0.69-2.51)	0.405
Medium ProS	83.86	1.53 (0.86-2.70)	0.146	107.59	**2.18 (1.13-4.21)**	**0.020**
High ProS	154.12	**2.54 (1.52-4.25)**	**<0.001**	171.42	**3.52 (1.92-6.42)**	**<0.001**
**High genetic risk**						
Low ProS	65.47	1.17 (0.71-1.92)	0.543	74.25	1.58 (0.85-2.94)	0.151
Medium ProS	76.80	1.30 (0.72-2.34)	0.384	120.81	**2.39 (1.26-4.52)**	**0.007**
High ProS	240.81	**3.95 (2.49-6.27)**	**<0.001**	232.49	**4.87 (2.76-8.59)**	**<0.001**

Abbreviations: ProS, protein risk score; PYs, person-years; HR, hazard ratio; 95% CI, 95% confidence interval.

The HR (95% CI) were estimated using Cox proportional hazard models. The adjusted covariates were age, gender, assessment center, ethnicity, occupation, Townsend deprivation index, BMI, smoking status, drinking status.

Table S6. Sensitivity analyses after excluding incident cases during the first year of follow-up.

Characteristics	N/Cases	Person-years	Model 0		Model 1		Model 2	
			HR (95% CI)	P value	HR (95% CI)	P value	HR (95% CI)	P value
**Low genetic risk**								
Low ProS	6855/43	91530.96	Reference		Reference		Reference	
Medium ProS	3248/26	42951.06	1.29 (0.79–2.09)	0.310	1.17 (0.71–1.92)	0.549	1.10 (0.67-1.81)	0.714
High ProS	3051/37	38986.67	**2.01 (1.30–3.12)**	**0.002**	1.86 (1.19–2.91)	0.006	**1.69 (1.08-2.65)**	**0.022**
**Medium genetic risk**								
Low ProS	6477/39	86797.68	0.96 (0.62–1.48)	0.841	0.97 (0.63–1.50)	0.906	0.94 (0.61-1.46)	0.785
Medium ProS	3222/35	42431.79	**1.75 (1.12–2.74)**	**0.014**	**1.69 (1.07–2.65)**	**0.023**	**1.60 (1.02-2.52)**	**0.040**
High ProS	3188/60	40653.11	**3.13 (2.11–4.63)**	**<0.001**	**2.95 (1.99–4.38)**	**<0.001**	**2.65 (1.77-3.95)**	**<0.001**
**High genetic risk**								
Low ProS	6182/52	82717.26	1.34 (0.89–2.00)	0.158	1.31 (0.88–1.97)	0.186	1.31 (0.87-1.96)	0.196
Medium ProS	3308/38	43773.85	**1.85 (1.19–2.85)**	**0.006**	**1.76 (1.13–2.73)**	**0.012**	**1.66 (1.07-2.59)**	**0.024**
High ProS	3503/101	44336.13	**4.83 (3.38–6.90)**	**<0.001**	**4.53 (3.16–6.49)**	**<0.001**	**4.14 (2.87-5.96)**	**<0.001**

Abbreviations: ProS, protein risk score; HR, hazard ratio; 95% CI, 95% confidence interval.

Model 0: adjusted for no covariates;

Model 1: adjusted for age, gender, assessment center, ethnicity, occupation, and Townsend deprivation index.

Model 2: adjusted for age, gender, assessment center, ethnicity, occupation, Townsend deprivation index, BMI, smoking status, drinking status.

Table S7. Sensitivity analyses after excluding incident cases during the first 3 years of follow-up.

Characteristics	N/Cases	Person-years	Model 0		Model 1		Model 2	
			HR (95% CI)	P value	HR (95% CI)	P value	HR (95% CI)	P value
**Low genetic risk**								
Low ProS	6821/38	91454.32	Reference		Reference		Reference	
Medium ProS	3220/18	42890.62	1.01 (0.58–1.77)	0.972	0.87 (0.49–1.56)	0.645	0.85 (0.47-1.52)	0.580
High ProS	2994/27	38874.11	**1.68 (1.02–2.74)**	**0.04**	1.51 (0.92–2.50)	0.105	1.44 (0.87-2.39)	0.155
**Medium genetic risk**								
Low ProS	6445/36	86725.46	1.00 (0.63–1.57)	0.994	1.02 (0.64–1.61)	0.941	0.98 (0.62-1.56)	0.944
Medium ProS	3192/30	42367.84	**1.71 (1.06–2.75)**	**0.029**	1.62 (1.00–2.63)	0.051	1.58 (0.98-2.58)	0.063
High ProS	3119/45	40517.25	**2.68 (1.74–4.12)**	**<0.001**	**2.48 (1.60–3.84)**	**<0.001**	**2.32 (1.49-3.62)**	**<0.001**
**High genetic risk**								
Low ProS	6148/42	82638.78	1.22 (0.79–1.90)	0.370	1.20 (0.77–1.86)	0.429	1.19 (0.77-1.85)	0.437
Medium ProS	3280/31	43717.15	**1.71 (1.06–2.74)**	**0.027**	1.60 (0.99–2.58)	0.055	1.56 (0.96-2.52)	0.071
High ProS	3413/72	44164.02	**3.94 (2.66–5.83)**	**<0.001**	3.62 (2.44–5.39)	**<0.001**	**3.48 (2.33-5.2)**	**<0.001**

Abbreviations: ProS, protein risk score; HR, hazard ratio; 95% CI, 95% confidence interval.

Model 0: adjusted for no covariates;

Model 1: adjusted for age, gender, assessment center, ethnicity, occupation, and Townsend deprivation index.

Model 2: adjusted for age, gender, assessment center, ethnicity, occupation, Townsend deprivation index, BMI, smoking status, drinking status.

Table S8. Sensitivity analyses after excluding participants with hypertension, diabetes, dyslipidemia or cardiovascular disease at baseline.

Characteristics	N/Cases	Person-years	Model 0		Model 1		Model 2	
			HR (95% CI)	P value	HR (95% CI)	P value	HR (95% CI)	P value
**Low genetic risk**								
Low ProS	4730/29	63570.35	Reference		Reference		Reference	
Medium ProS	1915/15	25553.95	1.29 (0.69-2.40)	0.430	1.18 (0.62-2.24)	0.607	1.11 (0.59-2.11)	0.748
High ProS	1583/28	20578.41	**2.97 (1.76-4.99)**	**<0.001**	**2.75 (1.63-4.66)**	**<0.001**	**2.50 (1.47-4.26)**	**0.001**
**Medium genetic risk**								
Low ProS	4409/33	59305.02	1.22 (0.74-2.01)	0.436	1.24 (0.75-2.04)	0.398	1.20 (0.72-1.98)	0.482
Medium ProS	1911/13	25433.57	1.12 (0.58-2.15)	0.736	1.11 (0.57-2.13)	0.762	1.05 (0.54-2.02)	0.894
High ProS	1634/27	21319.42	**2.76 (1.63-4.66)**	**<0.001**	**2.68 (1.58-4.53)**	**<0.001**	**2.45 (1.44-4.17)**	**0.001**
**High genetic risk**								
Low ProS	4295/38	57676.28	1.44 (0.89-2.34)	0.136	1.41 (0.86-2.29)	0.170	1.39 (0.86-2.26)	0.183
Medium ProS	1998/21	26678.57	1.72 (0.98-3.02)	0.058	1.60 (0.90-2.83)	0.106	1.44 (0.81-2.57)	0.219
High ProS	1808/52	23458.18	**4.83 (3.07-7.61)**	**<0.001**	**4.59 (2.91-7.26)**	**<0.001**	**4.16 (2.62-6.63)**	**<0.001**

Abbreviations: ProS, protein risk score; HR, hazard ratio; 95% CI, 95% confidence interval.

Model 0: adjusted for no covariates;

Model 1: adjusted for age, gender, assessment center, ethnicity, occupation, and Townsend deprivation index.

Model 2: adjusted for age, gender, assessment center, ethnicity, occupation, Townsend deprivation index, BMI, smoking status, drinking status.

Table S9. Sensitivity analyses in the original proteomic data without imputation.

Characteristics	N/Cases	Person-years	Model 0		Model 1		Model 2	
			HR (95% CI)	P value	HR (95% CI)	P value	HR (95% CI)	P value
**Low genetic risk**								
Low ProS	4958/34	66134.76	Reference		Reference		Reference	
Medium ProS	2376/23	31372.09	1.42 (0.84-2.41)	0.193	1.45 (0.85-2.47)	0.171	1.37 (0.80-2.33)	0.250
High ProS	2215/32	28080.58	**2.20 (1.36-3.56)**	**0.001**	**2.18 (1.33-3.57)**	**0.002**	**1.96 (1.19-3.22)**	**0.008**
**Medium genetic risk**								
Low ProS	4611/34	61820.54	1.07 (0.66-1.72)	0.781	1.06 (0.66-1.71)	0.795	1.02 (0.63-1.64)	0.94
Medium ProS	2271/33	29768.31	**2.15 (1.33-3.47)**	**0.002**	**2.13 (1.31-3.46)**	**0.002**	**2.02 (1.24-3.28)**	**0.005**
High ProS	2268/49	28819.99	**3.27 (2.11-5.07)**	**<0.001**	**3.36 (2.15-5.24)**	**<0.001**	**2.98 (1.89-4.68)**	**<0.001**
**High genetic risk**								
Low ProS	4462/36	59762.39	1.17 (0.73-1.87)	0.508	1.14 (0.71-1.83)	0.584	1.13 (0.71-1.81)	0.608
Medium ProS	2388/33	31482.6	**2.03 (1.26-3.28)**	**0.004**	**2.00 (1.23-3.25)**	**0.005**	**1.83 (1.12-2.98)**	**0.016**
High ProS	2548/76	32140.36	**4.55 (3.04-6.82)**	**<0.001**	**4.66 (3.08-7.04)**	**<0.001**	**4.22 (2.78-6.42)**	**<0.001**

Abbreviations: ProS, protein risk score; HR, hazard ratio; 95% CI, 95% confidence interval.

Model 0: adjusted for no covariates;

Model 1: adjusted for age, gender, assessment center, ethnicity, occupation, and Townsend deprivation index.

Model 2: adjusted for age, gender, assessment center, ethnicity, occupation, Townsend deprivation index, BMI, smoking status, drinking status.

Table S10. Outline of JoGH's Guidelines for Reporting Analyses of Big Data Repositories Open to the Public (GRABDROP) items

**JoGH guideline item**
1. Please list all papers published by each co-author in previous three years that were based on secondary analysis of a big data repository<br>**Response:**<br>1.Wang Y, Li Y, Tian T, Han R, Zeng N, Xie F et al. Genome-wide analysis of 3' untranslated region alternative polyadenylation quantitative trait loci identified a potential novel susceptibility locus for lung cancer in cross-ancestry populations. J Hum Genet. 2025;70:529-536.<br>2.Han R, Huang J, Zeng N, Xie F, Wang Y, Wang Y et al. Systematic analyses of GWAS summary statistics from UK Biobank identified novel susceptibility loci and genes for upper gastrointestinal diseases. J Hum Genet. 2023;68:599-606.<br>3.Tian T, Zeng J, Li YC, Wang J, Zhang DF, Wang DG et al. Joint effects of sleep disturbance and renal function impairment on incident new-onset severe metabolic dysfunction-associated steatotic liver disease. Diabetes Obes Metab. 2024;26:4724-4733.<br>4.Tian T, Hong T, Tian T, He Y, Wang X, Qian L et al. Association between ultra-processed foods consumption and systemic immune-inflammation biomarkers in US Adults: Cross-Sectional results from NHANES 2003–2023. Human Nutrition & Metabolism. 2025;42:1-9.<br>5.Tian T, Zhu L, Wu Z, Xuan W, Li Y, Fan J et al. Accelerated biological aging drives the progression from MASLD to cirrhosis. Metab Target Organ Damage. 2025;5:65.
2. Please explain the key elements of your study design and the use of the available datasets that make your study an original scientific contribution<br>**Response:**<br>This study used a large prospective cohort design based on UK Biobank participants with available plasma proteomic data, genetic data, and longitudinal follow-up for incident psoriasis. First, we used a large prospective cohort design with long-term

follow-up, which enabled the assessment of circulating proteomic signatures before the onset of psoriasis. Second, we systematically evaluated 2,919 plasma proteins and identified proteins associated with incident psoriasis risk after multiple testing correction and sensitivity analyses. Third, we integrated proteomic profiling with polygenic susceptibility to assess their combined contribution to psoriasis risk, an aspect that has not been well characterised in previous studies. Together, these design features provide new prospective evidence on circulating proteomic markers and their interplay with genetic susceptibility in psoriasis development.

3. Please list all publications that addressed similar research questions in the same dataset and indicate where you cited them in your paper

Response:

We have checked the relevant literature and confirm that publications addressing similar research questions using the UK Biobank dataset have been cited in our manuscript as references 19, 20, and 29.

4. Please explain how you addressed multiple testing through an appropriately rigorous statistical threshold and indicate this in the methods section

Response:

In the analysis, we tested 2,919 proteins; therefore, we controlled for multiple comparisons using the false discovery rate (FDR) method. Proteins with FDR-adjusted *P*-values <0.05 were considered statistically significant.

5. Please declare to what extent have AI chatbots been used in developing your paper and to which parts of the paper did they contribute.

Response:

AI chatbot tools were used only for language polishing and improving the clarity of expression in the revised manuscript.

Text S1. Explanation of authorship change statement

RZ participated in the verification and checking of research data and analytical codes and in revising the manuscript at the peer review stage. She sorted out relevant result records, and conducted comprehensive academic language polishing and grammatical revision. She thus contributed substantially to the accuracy of data analysis and the readability and standardisation of the manuscript. She, therefore, fulfilled the Journal's authorship criteria and, with signed consent from all authors, earned her position in the authorship byline. All authors have agreed to this change, and have provided manuscript versions and analytical code as proof of this change.
